# Gut-bone axis perturbation: Mechanisms and interventions via gut microbiota as a primary driver of osteoporosis

**DOI:** 10.1016/j.jot.2024.11.003

**Published:** 2025-01-21

**Authors:** Jingyuan Wei, Qi Liu, Ho-Yin Yuen, Avery Chik-Him Lam, Yuanyuan Jiang, Yuhe Yang, Yaxiong Liu, Xin Zhao, Long Xiao

**Affiliations:** a Translational Medical Innovation Center, Zhangjiagang Traditional Chinese Medicine Hospital Affiliated to Nanjing University of Chinese Medicine, Zhangjiagang, Jiangsu, 215600, China; b Department of Acupuncture and Moxibustion, Dongzhimen Hospital, Beijing University of Chinese Medicine, Beijing, 100700, China; c Department of Applied Biology and Chemical Technology, The Hong Kong Polytechnic University, Hung Hom, Hong Kong, China; d Jihua Laboratory, Foshan, Guangdong, 528000, China

**Keywords:** Bone metabolism, Gut microbiota, Osteoporosis, Therapeutic targets

## Abstract

A growing number of studies have highlighted the significance of human gut microbiota (GM) as a potential target for osteoporosis. In this review, we discuss the effect of GM to bone metabolism focusing on two aspects: the local alterations of the human gut permeability that modify how the GM interact with the gut–bone axis (e.g., intestinal leakage, nutrient absorption), and the alterations of the GM itself (e.g., changes in microbiota metabolites, immune secretion, hormones) that modify the events of the gut–bone axis. We then classify these changes as possible therapeutic targets of bone metabolism and highlight some associated promising microbiome-based therapies. We also extend our discussions into combinatorial treatments that incorporate conservative treatments, such as exercise. We anticipate our review can provide an overview of the current pathophysiological and therapeutic paradigms of the gut–bone axis, as well as the prospects of ongoing clinical trials for readers to gain further insights into better microbiome-based treatments to osteoporosis and other bone-degenerative diseases.

The translational potential of this article: This paper reviewed the potential links between gut microbiota and osteoporosis, as well as the prospective therapeutic avenues targeting gut microbiota for osteoporosis management, presenting a thorough and comprehensive literature review.

## Graphical abstract

**Figure:**
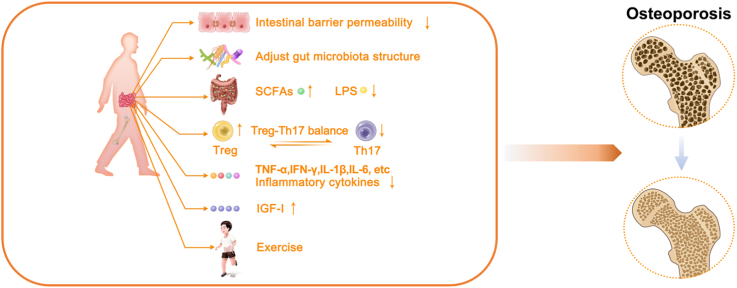

## 1 Introduction

Osteoporosis, characterized as a silent disease of the 21st century, has emerged as a significant public health concern due to its severe, chronic, and progressive characteristics. It is mainly recognized as a possible consequence of aging and affects postmenopausal women and elderly individuals [1]. The main symptoms of osteoporosis are back pain, spinal deformities and fractures. These symptoms arise from a decline in bone mineral density (BMD), which can be caused by the imbalance between bone formation and bone resorption (i.e., bone metabolism [2]), making osteoporosis a degenerative metabolic disease in nature. In the progression of osteoporosis, the activity of osteoclasts and osteoblasts is tightly coupled through multiple signaling pathways, ensuring a delicate equilibrium for bone metabolism [3]. Bone metabolism can be influenced by various factors including genetic, mechanical, vascular, nutritional, hormonal, and local factors (e.g., growth factors, matrix proteins, and cytokines) [4]. Given the comprehensive alterations in cell signaling cascades, it is inevitable that bone metabolism can be affected by distal organs, including the gut, which houses a vast array of microbes that can influence bone health through immunoregulation, inflammation, nutritional and hormonal factors [5,6].

Gut microbiota (GM) is highly metabolic and can be considered as a virtual endocrine organ with distal influence via the circulatory system [7]. Its role in the pathogenesis of osteoporosis, which has attracted more attention, can mainly be attributed to the systemic microbial metabolites produced in GM, and the secretion of these metabolites can be influenced by changes in the intestinal barrier, immune system, hormones, drugs and living habits. For instance, dysbiosis, the imbalance of bacterial composition, can cause an increase in intestinal barrier permeability and toxin releases, which further stimulates inflammatory responses and disrupts the bone remodeling pathways, resulting in osteoporosis [8]. It has also been suggested that dysbiosis can cause a decrease in BMD due to the increased inflammatory cytokines [9]. These associations between the pathophysiology of GM and osteoporosis indicate that GM could be a target of osteoporosis.

With the presence of many animal experiments and clinical trials (e.g., randomized controlled, cross-sectional, longitudinal) experimenting osteoporosis treatments via the gut–bone axis (e.g., diet intervention [10], fecal microbiota transplantation [11], probiotics and prebiotics [12], phytoestrogen [13]) in recent years, we believe that a comprehensive review documenting both the regulatory mechanisms and promising therapies for osteoporosis could provide novel insights for future directions of microbiome-based therapies, complementing other extensive reviews that focused only on the mechanisms of GM effects on immune system [14], inflammation [15], and bone degenerative diseases in general [16]. In our review, an overview of the physiology of osteoporosis and GM is first provided. We then proceed to summarize the mechanisms of the GM's effects on osteoporosis by dividing them into two directions: intestinal barrier permeability and GM-derived mediators. Finally, we document the current progress of preclinical experiments and clinical trials on the classified therapeutic targets above. We hope this review can provide new insights to improve, to combine, and even to propose new therapeutic strategies for clinical transformation and treatment.

## 2 Osteoporosis and GM

The physiology of osteoporosis and GM, their possible pathological origins, current consensus on the effectiveness of using GM in osteoporosis treatment will be presented, and their possible connections suggested.

### 2.1 Osteoporosis

Osteoporosis, which is chronic and progressive in nature, is characterized by decreased BMD and increased risk of fragility fractures. Clinically, T-score is used to express the risk of bone fractures by comparing an individual's BMD to that of a healthy adult of the corresponding age and race [17]. T-score is calculated by subtracting the reference mean BMD of the corresponding age and race group from the individual's measured BMD and dividing the result by the standard deviation of the reference population. A T-score of −2.5 or lower is a diagnosis of osteoporosis; a T-score between −2.5 and −1.0 is classified as osteopenia; and a T-score exceeding −1.0 means normal BMD. Osteoporosis can be divided into primary type and secondary type based on the factors that influence bone metabolism. In primary osteoporosis, BMD declines with increasing age. Primary osteoporosis can be further categorized into two types: postmenopausal osteoporosis (Type I) and senile osteoporosis (Type II). Postmenopausal osteoporosis is largely attributed to the decrease in estrogen levels following menopause, which leads to increased bone resorption by osteoclasts [18]. Senile osteoporosis affects both men and women in the later stages of life and is associated with the natural aging process. The pathogenesis of senile osteoporosis involves multifactorial elements including age-related decline in bone formation by osteoblasts, reduced sensitivity to anabolic hormones, and increased inflammation, all culminating in a negative impact on bone remodeling [19]. Secondary osteoporosis refers to the condition that low bone mass and an increased risk of fractures occur as a result of factors other than aging and menopause. These factors, which include various conditions, diseases, and prolonged use of drugs, can directly or indirectly affect bone metabolism [20].

Osteoporosis can be explained from the perspective of altered maintenance of normal bone mass. During the normal bone renewal process, a dynamic interplay occurs between bone formation and bone resorption. At a cellular level, osteoclasts promote bone resorption, while osteoblasts enhance bone formation. Both types of bone cells work in certain cell units called basic multicellular units (BMUs) [21]. However, in osteoporosis, there is an imbalance between bone renewal and bone resorption due to the increase in osteoclast activity with increasing bone resorption and a corresponding decrease in bone formation (i.e., bone resorption level is greater than bone formation level) [22]. Besides, secretory factors originating from bone cells (osteoclasts, osteocytes and osteoblasts) also undergo changes during osteoporosis. For example, activated osteoclasts promote bone resorption by dissolving bone minerals and degrading bone tissue through the secretion of proteolytic enzymes (cathepsin K and MMP9) and hydrochloric acid. Osteocytes suppress bone formation by releasing sclerostin, Dickkopf-related protein 1, and Frizzled-related protein 1. Osteocytes and Osteoblasts can also produce RANKL to activate RANK-RANKL interaction and increases the expression of genes associated with osteoclastogenesis [23]. The current understanding of the mechanisms underlying osteoporosis suggests that a defective coupling of bone formation and bone resorption contributes to the gradual reduction of BMD, which is the basic pathological mechanism. Although the exact cause of the defective coupling in osteoporosis is not fully understood, it is believed to be a common aspect in age-related diseases that we can attribute to a general accumulative and degenerative process [24].

### 2.2 Gut microbiota (GM)

The vast majority of microbes (10–100 trillion) in the human body are found in the gastrointestinal tract, with most of them located in the distal gut [25]. The term “microbiota” refers to a complex community of microorganisms that plays a pivotal role in various systemic functions. These include the absorption of nutrients, the preservation of metabolic homeostasis, the maturation of the immune system, and defense against infections [26]. The extent of GM functions and its dysbiosis influence a diverse set of chronic and noncommunicable clinical diseases beyond its anatomical proximity, including inflammatory bowel disease [27], obesity [28], metabolic disease [29], malnutrition [30], nervous system disease [31], therioma [32], and cardiovascular disease [33]. Mechanistically, the function of GM is governed by the composition, diversity, and abundance of the intestinal bacterial species. Key components that influence the balance of microecology are *Firmicutes, Bacteroidetes, Actinobacteria,* and *Proteobacteria*. The *Firmicutes*/*Bacteroidetes* ratio, which has been demonstrated to be negatively correlated with bone volume, is an example of clinical markers that can be derived from its composition to measure GM dysbiosis [34]. When the GM becomes disrupted such as in form of an imbalanced *Firmicutes/Bacteroidetes* ratio, the integrity of the intestinal barrier would be compromised, leading to microbiota translocation and the occurrence of “intestinal leakage” where permeated bacterial byproducts trigger immune responses, affecting distant organs such as bone [35,36]. In addition, both animal experiments and patient data demonstrated pathological changes in the diversity, abundance, and metabolic pathways of the GM in osteoporosis patients. For example, patients with primary osteoporosis had a higher level of *Firmicutes* phyla, *Lachnoclostridium* and *Kelbsiella* genera as well as a lower level of *Bacteroidetes* phyla and *Prebotella* genera than normal controls in a diversity analysis [37]. In another research, GM richness and diversity were also reduced in postmenopausal osteoporosis patients, and metabolite indicators demonstrated that higher levels of N-acetylmannosamine, histamine, adenosine, deoxyadenosine, L-lysine and L-threonate were found in the postmenopausal osteopenia and osteoporosis patients than normal controls [6]. Changes in GM diversity and metabolite further affect SCFAs production, inflammation and immune regulation.

### 2.3 Current consensus on GM in the treatment of osteoporosis

Current understanding and recommendations on microbiome-based therapies remain ambiguous as there is not yet any consensus on what constitute a healthy and pathological GM given the presence of individual differences, regional differences and other factors [38]. For this reason, current consensus consider conservative treatments such as dietary intervention and substance supplements as standard strategies to both gradually investigate the alterations in GM (e.g., composition, activities) and to address clinical needs (restoring to a healthy GM) [39]. The strategies include how increased calcium (Ca) intake by vitamin D can reduce bone mineral loss with changes in abundance of specific bacteria [40], how probiotics and prebiotics can regulate Ca metabolism to target bone disorders [41], and how antibiotics and mucus supplements can prevent trabecular bone loss and may improve glucocorticoid-induced osteoporosis [42].

## 3 Mechanisms of the GM's effects on osteoporosis

**Fig. 1:**
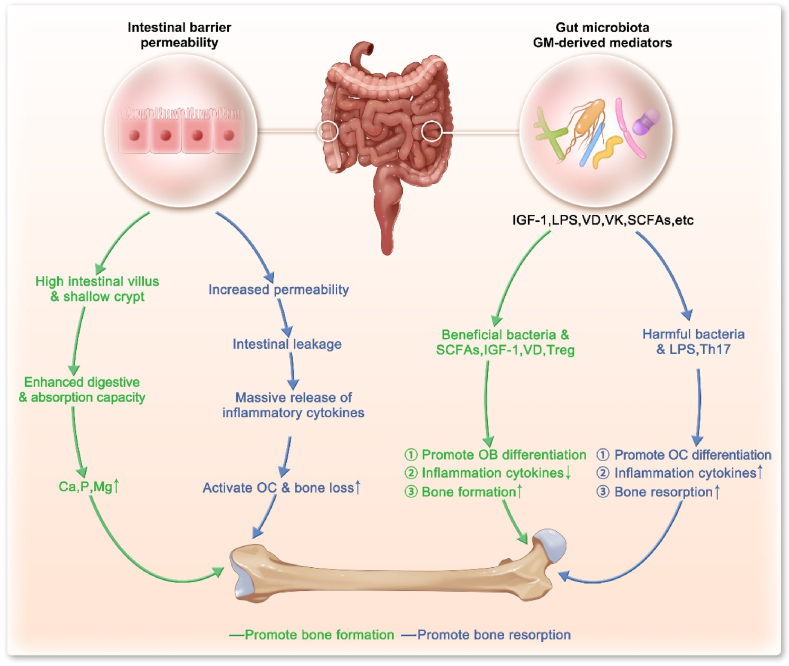
**Mechanism of the intestinal barrier and GM-derived mediators that affect bone metabolism.** The intestinal barrier and GM-derived mediators mainly regulate the changes in BMD through the balance between bone formation and bone resorption. GM, gut microbiota; LPS, lipopolysaccharide; OC, osteoclasts; OB, osteoblasts; SCFAs, short-chain fatty acids; IGF-1, insulin-like growth factor 1; Treg, regulatory T cell; Th17, T helper 17 cell.

Emerging research has demonstrated that a diverse set of mechanisms is involved in GM's regulation of the bone metabolism and bone homeostasis [43]. These mechanisms can be categorized into two major parts: intestinal barrier permeability and GM-derived mediators (Fig. 1).

### 3.1 Intestinal barrier permeability

Intestinal barrier regulates the transport of molecules between the intestinal lumen and the submucosa [44,45]. Under normal physiological conditions, the intestinal barrier – the gaps between intestinal epithelial cells – can selectively allow passage of molecules, nutrients, and smaller proteins with a molecular diameter smaller than around 8 Å [46]. Under pathological conditions, there is a heightened permeability in the intestine that allows larger molecules and potential antigens with a diameter of approximately 100 Å to traverse through the barrier [46]. The invasion of undesirable molecules may trigger abnormal inflammatory responses in the gut and lead to elevated levels of proinflammatory factors, which in turn would accelerate the progression of osteoporosis [47]. Recent studies have categorized the causes of intestinal barrier permeability into two main types: biochemical alterations and structural impairments.

#### 3.1.1 Impairment of the intestinal structure

The intestinal structure affects the manner in which molecular transport occurs across the intestinal lumen. The intestinal villus height (VH) and crypt depth (CD) are two critical phenotypic characteristics associated with the digestive and absorptive capacity of the intestine [12]. The villus height refers to the protrusions or finger-like structures on the inner surface of the intestinal lining [48], while crypt depth refers to the invaginations or grooves between the villi. Together, the VH and CD contribute to the overall surface area available for nutrient absorption and play a key role in optimizing the efficiency of digestion and absorption within the intestine [49,50]. Meanwhile, a normal VH/CD ratio is more conducive to the growth and colonization of beneficial bacteria. Mechanistically, the effect of intestinal structure on bone metabolism is affected by absorption efficiency [51]. When absorption efficiency decreases, there is a reduced intake of Ca, magnesium (Mg) and phosphorus (P), which are the basic elements that make up bones. The lack or insufficiency of these three elements can lead to bone metabolic imbalance [52].

#### 3.1.2 Impairment of the intestinal barrier induced by biochemical alterations

Since changes in intestinal barrier permeability are physical in nature, alterations such as those in the tightness of the intestinal barrier represent the major factors that impair GM metabolism. Various tight junction proteins, such as occludin, claudins, connexins, and zonula occludens 1 (ZO-1), are involved in maintaining the integrity of the intestinal barrier [53]. The impact of biochemical changes caused by gut microbiota on intestinal barrier permeability is bidirectional. Aging has been reported to affect intestinal barrier permeability in humans. In a cross-sectional analysis, participants were divided into three groups based on age: the young (aged 7–12), the adult (aged 20–40), and the old (aged 67–77). The result indicated that the old-aged group had the highest intestinal barrier permeability, and that the aging gut was characterized by higher levels of cytokine interleukin (IL)-6, which in part affected claudin 2 [54]. Additionally, chemical or surgical menopause in animal models were demonstrated to downregulate claudin and junctional adhesion molecule 3, leading to impaired intestinal barrier function [55]. A pilot study on 65 women demonstrated that intestinal barrier permeability increased from pre-to post-menopause with decreased estradiol and increased follicle stimulating hormone (FSH). The increased intestinal barrier permeability led to higher expression of inflammation marker (high-sensitivity C-reactive protein) and lower BMD [55]. These results provided some evidence for the role of menopausal transition and sex hormones in maintaining the tightness of the intestinal barrier. Changes in intestinal barrier permeability can also happen indirectly, as demonstrated by Schepper et al. in their experiments, which showed that antibiotic intervention led to the dysbiosis of mice GM, which significantly enhanced their intestinal barrier permeability to result in trabecular bone destruction, bone loss, and ultimately osteoporosis [56]. Mechanistically, the systemic effect of intestinal barrier permeability on bone metabolism can be attributed to gastrointestinal immunity [57,58]. Specifically, the increase in intestinal permeability can result in the occurrence of “intestinal leakages,” leading to abnormally high levels of inflammatory cytokines. This systemic inflammation in turn can stimulate the activation of osteoclasts, consequently accelerating bone degradation [59,60]. However, the upregulation of GM metabolites can reinforce the intestinal barrier, thus reducing its permeability. Butyrate (GM metabolites), notably, stimulates an increase in mucus production by enhancing Mucin-2 and SPDEF (goblet cell marker genes) in macrophages/goblet-like cells through the M2 macrophage-associated WNT/extracellular signal-regulated kinase (ERK) signaling pathway [61]. The upregulation of butyrate can also modulate the expression of synaptopodin, an actin-associated protein integral to the mechanical barrier's strength and cell motility [62]. The upregulation of other GM metabolites (indole-3-ethanol, indole-3-pyruvate, and indole-3-aldehyde) also contributes to the enhancement of the intestinal barrier by modulating apical junctional complex (myosin IIA and ezrin) and reducing inflammation [63]. These metabolites collectively provide a protective mechanism against increased intestinal permeability and support the intestinal epithelium's overall health.

### 3.2 GM-derived mediators

GM-derived mediators are mainly metabolites that affect bone metabolism through their interacting with the immune system and endocrine system [64]. In the following sections, we will summarize the effect of GM metabolites, alterations in immunoregulation, and alterations in endocrine regulation on osteoporosis.

#### 3.2.1 Dysregulation in GM metabolites

A vast majority of GM metabolites have been shown to be closely involved in the maintenance of bone formation and bone resorption balance [65]. Therefore, the exploring of the generation, the associated metabolic pathways, and the functions of these GM metabolites shall provide new avenues for developing new targeted therapeutic and intervention approaches for osteoporosis.

Short-chain fatty acids (SCFAs) are one of the most studied GM metabolites that have been shown to have association with chronic diseases via systemic inflammation [66]. SCFAs constitute primarily of carboxylic acids characterized by short carbon chains that typically range from 1 to 6 atoms. These compounds are generated as a result of the fermentation process of indigestible carbohydrates, particularly dietary fiber, by GM [67]. The type and content of the produced SCFAs are also determined by the microflora composition. The major components of SCFAs are butyrate, propionate, and acetate, which make up of nearly 80 % of all SCFAs; acetate can be produced by common intestinal bacteria, while propionate and butyrate are only produced by a select group of bacteria. Butyrate is produced by *Faecalibacterium prausnitzii*, *Eubacterium rectale*, *Eubacterium hallii*, *Anaerostipes hadrus*, and *Coprococcus catus*. Propionate is produced by *Bacteroides uniformis*, *Bacteroides vulgatus*, *Prevotella copri*, and *Phascolarctobacterium succinatutens* [68]. All these SCFAs have been reported to influence bone density through the inhibition of osteoclast differentiation, the promotion of osteoblast differentiation, calcium absorption, and anti-inflammation [[69], [70], [71], [72]]. For example, propionate and butyrate have been shown to change the metabolic state of pre-osteoclasts and subsequently suppress the expressions of osteoclast genes like TRAF6 and NFATc1 in cell culture experiments. This inhibition of gene expression subsequently hampers osteoclastogenesis and reduces bone resorption, ultimately creating a conducive environment for osteoblasts and facilitating bone formation [72]. It has also been demonstrated that by treating IFN-γ-induced RAW 264.7 cells with sodium butyrate, sodium phenylbutyrate and sodium phenylacetate, which are three main types of SCFAs, the activity of nuclear factor kappa-B (NF-κB) and ERK pathways can be inhibited to reduce autoimmune inflammation [73]. In addition, SCFAs lower the pH of the intestinal cavity, which can be helpful to improve the solubility of minerals and improve the absorption efficiency of calcium. SCFAs can also indirectly regulate hormone levels, and supplementation with SCFAs can increase the level of IGF-1 in serum to indirectly affect bone metabolism. SCFAs bind G protein-coupled receptors to promote the proliferation and differentiation to inhibit osteoclast differentiation [74].

Bile acid is another important GM metabolite that participates in the gut–liver axis. Primary bile acids form the bile salts in liver, which are secreted into the small intestine, and metabolized by GM to produce secondary bile acids. GM can alter the quantity and type of primary bile acids, and then result in different metabolic effects. Secondary bile acids can also serve as ligands to participate in bone metabolism. Current research has shown that bile acids function as ligands for receptors including farnesoid X receptor (FXR), G protein-coupled bile acid receptor 5 (TGR5), and vitamin D receptor [75]. An *in vivo* study demonstrated that FXR-deficient male mice had a decrease of 4.3 %–6.6 % in bone mass at 8–20 weeks of age compared to normal male mice [76]. Cell experiments have also shown that chenodeoxycholic acid (CDCA) and 6α-ethyl-chenodeoxycholic acid (6-ECDCA) as natural and semisynthetic bile acid are strong agonists of FXR that can activate FXR to upregulate Runt-related transcription factor 2 (Runx2) and promote osteoblast formation through ERK and β-catenin signaling pathways [77]. As one of the receptors of bile acid, TGR5 has recently been found to express in osteoblast-like cell line MC3T3-E1 cells. An *in vitro* study demonstrated that the activation of TGR5 could promote the expression of Runx2 and osteogenic-related proteins (e.g., alkaline phosphatase (ALP), osteocalcin (OCN), osterix (OSX)) through adenosine monophosphate-activated protein kinase (AMPK) signaling pathway [78]. It has also been suggested that dual activation of TGR5 and FXR can enhance osteoblastogenesis more effectively than activating FXR or TGR5 alone [79]. Moreover, secondary bile acids produced by GM have been proved to decrease the level of ROS and inflammatory reaction by reducing the TNF-α-induced immune response, which demonstrated the potential role of these metabolites in the development and progression of osteoporosis [80].

Vitamin K is another frequently studied GM metabolite that can be synthesized by GM. Natural vitamin K exists mainly in two biologically active forms: vitamin K1 and vitamin K2. Vitamin K1 is present in plant margarine and vegetables., while vitamin K2 consists of a group of menaquinones (MK-n, varies from MK-4 to MK-13) that can be synthesized by GM. For example, MK-6 can be synthesized by *Eubacterium lentum*, MK-7 by *Veillonella*, MK-8 by *Escherichia coli*, as well as MK-10 and MK-11 by *Bacteroides species* [81]. Recent research has demonstrated that vitamin K supplementation can decrease bone resorption markers in ovariectomized (OVX) mice [82]. Vitamin K regulates bone remodeling by promoting the osteoblast-to-osteocyte transition and by limiting osteoclastogenesis., which mainly depends on activating bone-related proteins such as OCN [83], and matrix Gla protein (MGP) [84]. Trimethylamine N-oxide (TMAO), another metabolite that can be produced by GM, is originated when GM metabolizes choline to produce trimethylamine (TMA), which is then oxidized in the liver to form TMAO [85]. TMAO can impede the differentiation of bone marrow mesenchymal stem cells (MSCs) into osteoclasts through the NF-κB pathway [86]. Many other GM metabolites participate in bone metabolism indirectly via the repair of intestinal barrier permeability. For example, indole derivatives can stimulate the production of antimicrobial peptides, mucous proteins, and enhance the proliferation of intestinal villus cells, all of which help preserve the integrity of the intestinal mucosa [87]. Polyamines play a role in mediating the proliferation of intestinal epithelial cells to maintain the function of the intestinal barrier; they can also regulate bone metabolism through the effect on immune system [88]. Extracellular vesicles (EVs) derived from bacteria with spherical lipid bilayer nanostructures are another GM metabolite. The diameters of EVs range from 10 to 400 nm, containing a variety of components, including bioactive proteins, lipids, nucleic acids, and virulence factors. Their unique nanoscale structure enables efficient long-distance transport of EVs and their internal molecules, ensuring they reach intracellular compartments in a concentrated, protected, and targeted way. For example, bacterial EVs derived from the intestinal strain *Proteus mirabilis* have been reported to prevent bone resorption. These bacterial EVs can influence mitochondrial function and apoptotic pathways by miR96–5p/Abca1 to inhibit osteoclastogenesis. *Proteus mirabilis*-derived EVs have also been proved to reduced bone loss in OVX mice [89]. Engineered EVs with enhanced targeting capability have been developed to treat osteoporosis [90].

#### 3.2.2 Alterations in immunoregulation

The term “osteoimmunology”, which was coined in the year 2000, encompasses the current understanding of the reciprocal interactions and regulation between the skeletal and immune systems. Interestingly, different members of GM are also involved in this regulatory process and play their respective roles.

Bacterial components (e.g., lipopolysaccharide (LPS), peptidoglycan (PGN), flagellin) can participate in the regulation of bone metabolism through the innate immune system [91]. On the one hand, these components are crucial building blocks of bacterial cell walls, safeguarding bacteria against antimicrobial agents [92]. On the other hand, they function as the primary source of bacterial toxins when they are released from the outer membrane of deceased bacteria [93]. When the permeability of the gut is compromised, certain bacterial components called pathogen associated molecular patterns (PAMPs), can enter the circulation to activate pattern recognition receptors (PRRs) of intestinal epithelial cells and bone cells (e.g., osteoclasts, osteoblasts) [94]. Macrophages and dendritic cells, which are a part of the innate immune response in GM, also possess PRRs, and their activation can also lead to excessive inflammation, which influences the process of bone absorption and formation [95]. For instance, most signals of toll-like receptor (TLR), one of the common PRRs, are transmitted through the MyD88 protein, which in turn activates the mitogen activated protein kinase (MAPK) and NF-κB pathways [96]. TLR 4 and TLR 5 are the innate immune receptor for LPS and flagellin respectively, and for bone formation, osteoblasts can respond to TLR 4 and TLR 5 ligands and generate pro-inflammatory cytokines (e.g., IL-1β, IL-6, TNF-α), which activate macrophages (the precursor cells of osteoclasts). In addition, there are several modes of actions for TLRs depending on the stage of osteoclast differentiation. During the early bone remodeling stage, the binding of microbial TLR ligands to TLR 4 in preosteoclasts can lead to the suppression of osteoclast differentiation. This suppression occurs through IL-12-mediated inhibition of RANKL-induced osteoclast differentiation [64]. During the late bone remodeling stage, the activation of TLR induced by LPS facilitates the survival of osteoclasts through the rapid degradation of I-κB in osteoclasts. However, mice that were deficient in the TLR4 gene were not able to support osteoclast survival when activated by 10.13039/501100012274LPS. Different from macrophages, those pro-inflammatory cytokines (e.g., IL-1β, TNF-α) were less abundant in osteoclastic microenvironment when induced by LPS [97]. In addition to LPS and flagellin, PGN has also been found to activate innate immune receptors. Specifically, PGN exerts its biological activity by interacting with nucleotide oligomerization domain (NOD)-like receptor (NLR), which is another common PRR. NOD1 and NOD2 are two important members of the NLR family and are recognized as natural immune receptors for PGN. When PGN binds to NOD1 or NOD2, the initiated signals are mediated through the receptor-interaction protein (RIP2), which in turn activates NF-κB pathway. It has also been demonstrated that the regulation of GM on TNF-α and osteoclastogenesis (e.g., RANKL, CSTK) is dependent on NOD1 and NOD2 signaling pathways [98].

**Fig. 2:**
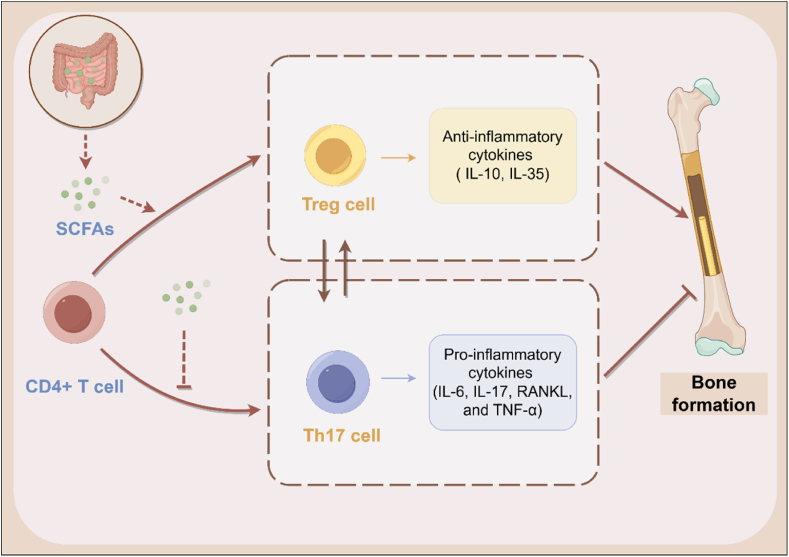
**The effect of GM on CD4**^**+**^**T cells in bone metabolism.** SCFAs secreted by GM regulate the Treg-Th17 balance to promote bone formation through decreasing the number of Th17 cells and the production of pro-inflammatory cytokines, increasing the number of Treg cells and the generation of anti-inflammatory cytokines.

Several immune cell types (e.g., T cells, B cells, dendritic cells, macrophages), cytokines and antibodies in the adaptive immunity system are also involved in osteoporosis [99], but evidence for their roles in the gut–bone axis has not been well established. Recent studies revealed that germ-free (GF) C57BL/6 mice exhibited increased bone mass associated with decreased amount of CD4^+^ T cells and osteoclast precursor cells in bone marrow compared with conventionally raised (CONV-R) C57BL/6 mice. However, bone mass as well as the number of CD4^+^ T cells and osteoclast precursor cells normalized after colonization of normal GM from CONV-R mice in GF mice, which provided evidence of GM-induced changes in adaptive immune system and bone mass [100]. GM also contributes to the activation, polarization and function of CD4^+^ T cells via cytokines (Fig. 2) [101]. Among T cell subpopulations, the balance between Th17 cells and Treg cells has been suggested to profoundly impact the progression of osteoporosis [102]. Treg cells express CTLA-4, which can activate indoleamine-pyrrole 2,3-dioxygenase (IDO), causing apoptosis of osteoclasts [103]. Besides, cytokines secreted by Treg cells (e.g., TGF-β, IL-10, IL-35) can regulate bone formation via MAPK and Smad-related proteins [104,105], and upregulate osteoprotegerin (OPG) and downregulate cytokines (e.g., RANKL, M-CSF) to inhibit the differentiation of osteoclasts [106]. By contrast, Th17 cells express RANKL, which can induce the generation of RANK to promote differentiation of osteoclast precursor cells into osteoclasts [107]. Cytokines secreted by Th17 cells (e.g., IL-17, IL-22, IFN-γ) can directly regulate bone resorption [108]. Moreover, Th17 cells can induce macrophages to produce pro-inflammatory factors (e.g., TNF-a, IL-1, IL-6) to indirectly promote bone resorption [109]. Osteoimmune disorders caused by imbalance between Th17 cells and Treg cells, as well as cytokines are important triggers of osteoporosis [110]. Since both of Th17 and Treg cells originate from CD4^+^ T cells, they can undergo reciprocal conversion with cytokines, which precisely regulate the immune response in osteoporosis. In OVX rats, there were an increase in the number of Th17 cells and a decrease in the number of Treg cells. After 12 weeks of treatment with Chinese medicine extract granules, probiotics could be enriched to generate more SCFAs, which further increased the number of Treg cells and related cytokines to restore the Th17-Treg balance for bone formation [110]. Supplementation of probiotics (e.g., *Lactobacillus fermentum* [111], *Lactobacillus rhamnosus* [112]) has also shown to increase the number of Treg cells and related cytokines in animal experiments. This effect has also been demonstrated to be mediated by the production of SCFAs. Apart from Th17-Treg balance, immunoglobulin A (IgA) also plays a role in osteoporosis. IgA production is stimulated by various cytokines (TGF-β, IL-4, IL-10, IL-5, IL-6) and immune cells (M cells, dendritic cells, T cells, B cells). IgA can enhance the functionality of the mucosal barrier, thereby facilitating colonization, regulating metabolism, and preventing systemic inflammation [113].

#### 3.2.3 Alterations in endocrine regulation

It has been suggested that the intestine can be viewed as one of the most important endocrine organs in the human body [7]. In this context, hormones are the major regulators for osteoporosis [114]. Specifically, the intestine can directly generate hormones that enter the bloodstream to influence distal sites. The decrease in hormone levels (e.g., estrogen) can also lead to alterations in GM structure, reduction of GM metabolites, and impairment of the intestinal barrier [55].

Insulin-like growth factor 1 (IGF-1) is a key regulator that can affect bone formation by promoting osteoblast-to-osteocyte transition through the IGF-1/IGF-1R pathway and promote the differentiation of MSCs into osteoblasts through the IGF-1/PK13/mTOR pathway [115]. In one study, specific-pathogen-free (SPF) C57BL/6 mice exhibited blunted bone formation, reduced osteoblastic cell differentiation and mineralization. In addition, there was a suppression in the expression of Igf1 in bone tissue and a decreased serum IGF-1 levels in SPF mice compared with GF mice. The results demonstrated that the commensal microbiota's anti-osteoblastic actions were potentially mediated via suppression of local IGF1-signaling in skeletal tissues [116]. In another study, suppressed serum IGF-1 levels and inhibited bone formation were induced by treating mice with antibiotics (i.e., ubiquitous changes to the GM). However, supplementation with SCFAs (GM metabolite) in these mice restored the IGF-1 level and bone mass, demonstrating the interplay between IGF-1, GM health, and bone health [16].

Serotonin, also known as 5-hydroxytryptamine (5-HT), is a hormone and neurotransmitter that plays an important role in bone metabolism. The production of 5-HT primarily takes place in the gut, which accounts for approximately 95 % of the total amount of 5-HT in the body, while the rest is synthesized in the central nervous system [117]. Since 5-HT is unable to pass through the blood brain barrier (BBB), studies on the effect of 5-HT on bone metabolism mainly focus on it as a GM hormone. Peripheral serotonin acts on the 5-HT receptor on both osteoclasts and osteoblasts. The 5-HT receptors on osteoclasts are known as 5-HT1 and 5-HT2, which can bind to 5-HT to increase bone resorption; whereas the 5-HT receptors on osteoblasts are known as 5-HT2B and 5-HT7, which can bind to 5-HT to affect the proliferation of osteoblasts [118], though the exact cellular mechanisms activated by 5-HT remain unknown. It has also been suggested that peripheral serotonin might inhibit LPS-induced secretion of pro-inflammatory cytokines, which can indirectly affect bone metabolism. Although 5-HT cannot traverse through the BBB, brain-derived 5-HT can activate 5-HT2C receptors on neurons to decrease sympathetic outputs. This reduction in sympathetic activity further promotes bone formation and inhibits bone resorption [119].

Parathyroid hormone (PTH), a product of the parathyroid glands, is located behind the thyroid in humans. PTH is a critical regulator of skeletal development and postnatal skeletal maturation due to its capacity to regulate vitamin D and serum calcium [120]. PTH can stimulates the synthesis of vitamin D in the kidney by 1α-hydroxylase, which will in turn lead to increased Ca^2+^ absorption from the gut [121]. PTH acts on PTH receptor 1 to significantly increase initial bone formation and subsequently induce bone resorption markers to increase a later bone remodeling as the anabolic window. PTH, PTH analogs, and intermittent administration of PTH have been employed to treat osteoporosis. Current research has also demonstrated that butyrate (GM metabolite) is required for PTH to stimulate bone formation and increase bone mass. Specifically, butyrate potentiated the capacity of (PTH) to induce the differentiation of native T cells into Tregs, thus enhancing the release of Wnt10b from CD8^+^ T cells to stimulate bone formation [122].

In addition to the hormones mentioned above, many other hormones also take part in the gut–bone axis (e.g., glucagon-like peptide-1, gastric inhibitory polypeptide, ghrelin). Although they have been investigated in some recent preclinical studies [123,124], there is still a lack of sufficient preclinical and clinical data to clarify their mechanisms in osteoporosis. It is important to note that there is a complex interplay and crosstalk between and among different hormones and organs. Therefore, whether other hormones play an important role in bone metabolism as well remains to be investigated.

## 4 Potential therapeutic strategies for osteoporosis

Pharmacological treatment is the commonly used treatment regimen for osteoporosis. A variety of agents have been utilized for the treatment of osteoporosis, including bisphosphonates, calcitonin, estrogen, parathyroid hormone, selective estrogen receptor modulators (SERMs). However, these drugs often lead to varying degrees of side effects. For example, common side effects of bisphosphonates include stomach upset and heartburn. Although rare, serious complications can occur, such as atypical femoral fractures and osteonecrosis of the jaw [125]. Moreover, long-term use of drugs like estrogen and calcitonin might result in an elevated susceptibility to malignancy. There is growing interest in alternative approaches that may offer benefits with fewer risks. GM can not only significantly influence bone metabolism but also minimize the adverse effects associated with traditional osteoporosis treatments.

**Fig. 3:**
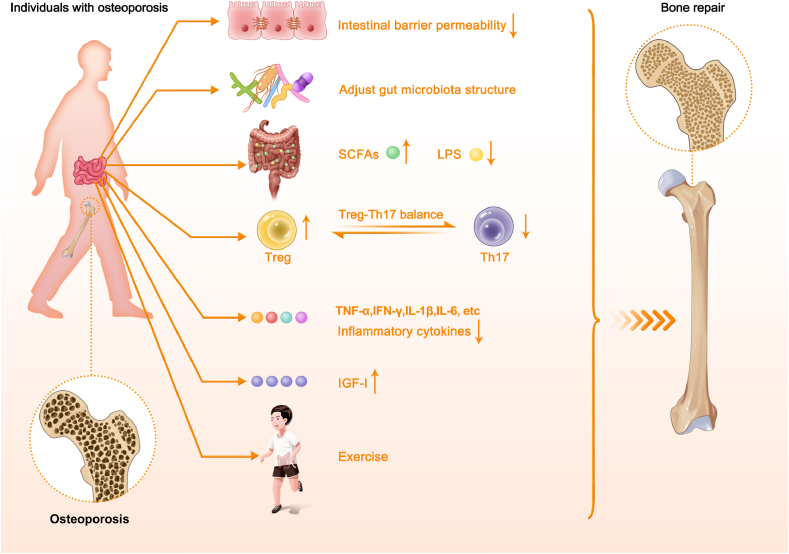
**A schematic diagram of potential therapeutic targets for osteoporosis associated with GM.** GM can be targeted for the treatment of osteoporosis through reducing the intestinal barrier permeability, adjusting the GM structure, increasing the concentration of SCFAs, reducing the concentration of LPS, regulating Treg-Th17 balance, reducing the level of inflammatory factors (e.g., TNF-α, IFN-γ, IL-1β, IL-6), increasing serum IGF-1 levels, and doing regular exercise. SCFAs, short-chain fatty acids; LPS, lipopolysaccharide; Treg, regulatory T cell; Th17, T helper 17 cell; TNF-α, tumor necrosis factor alpha; IFN-γ, interferon γ; IL-1β, interleukin 1β; IL-6, interleukin 6; IGF-1, insulin-like growth factor 1.

Researchers determine targets for osteoporosis treatment by addressing and rectifying dysfunctional mechanisms [126]. Based on our current knowledge on the mechanisms of GM-mediated bone metabolism, various targets identified for osteoporosis are summarized in Fig. 3. From the perspective of the local GM microenvironment, the reduction of intestinal barrier permeability can prevent intestinal leakages and enhance the absorption of Ca, vitamin D, and other nutrients to promote bone formation. Adjusting GM composition by improving the type, diversity and abundance of GM can also be beneficial to osteoporosis. In addition, the impact of GM-derived mediators, including SCFAs, LPS, Treg-Th17 balance, inflammatory cytokines, and hormones, can be effectively regulated to participate in bone metabolism. Although there are already various targets for the regulating of osteoporosis, effective treatments are still unavailable for various reasons. Firstly, the composition and function of GM can vary significantly from person to person, making it difficult to identify specific microbial factors contributing to bone diseases [127]. Secondly, individual responses to microbiome-based treatments can vary widely, factors such as host genetics, immune status, and overall health can influence treatment outcomes, making it difficult to predict and optimize the response in each patient. Thirdly, translating research findings from preclinical studies to clinical applications is a complex process. Validating the effectiveness and safety of potential treatments in human clinical trials requires rigorous scientific investigation for osteoporosis [128].

Clinical applications of microbiome-based therapies include dietary intervention, probiotics and prebiotics, fecal microbiota transplantation and the use of antibiotics [129]. Among them, the long-term use of antibiotics is limited despite their effectiveness in clearing deleterious and pathogenic microbes due to the adverse side effects such as reduced diversity and richness of intestinal species [130], as well as the emergence of drug resistance [131]. Considering the shortcomings of antibiotics, our review primarily focuses on the other three promising microbiome-based therapies, along with conservative treatments such as exercise, for the treatment of osteoporosis. The details of these therapies are summarized in the following subsections and outlined in Table 1.

**Table 1:** Targets and mechanisms of the gut microbiota for the treatment of osteoporosis (animal experiment).

**Target/Intervention**	**Model**	**Duration**	**Results and related mechanisms**	**Ref.**
**Probiotics and prebiotics**	Post-ABX (antibiotic-treated) mice were supplemented with sterile water (n = 9), with *Lactobacillus reuteri* (n = 10), *Lactobacillus rhamnosus* GG (n = 4), with nonpathogenic *Escherichia coli* (n = 5)	4 weeks	*Lactobacillus reuteri* can delay the disruption of the intestinal epithelial barrier, decrease the *Firmicutes/Bacteroidetes* ratio and reduce bone loss of the femur and vertebral trabeculae.	[56]
**Probiotics**	OVX mice directly supplemented with *Akk* twice a week (n = 5)	8 weeks	*Akkermansia muciniphila-*derived EVs have been designed to directly target bone cells, promoting osteogenesis and inhibiting osteoclastogenesis to preserve bone mass and strength.	[164]
**Probiotics**	8-week-old female C57BL/6 mice were randomly divided into four groups: Sham, OVX, OVX treated with *LGG*-EVs, and OVX treated with BT-*LGG*-EVs	8 weeks	*Lactobacillus rhamnosus* GG-EVs membranes in a recent study, which can deliver intrinsic miRNAs to bone, thus enhancing osteoblast mineralization.	[165]
**FMT therapy**	OVX mice using the fecal donors of healthy mouse through gavage every day (n = 5)	8 weeks	FMT therapy can promote bone formation by inhibiting pro-osteoclastogenic cytokines like TNF-α and IL-1β.	[167]

### 4.1 Dietary intervention

Dietary intervention has been employed to mediate osteoporosis and its therapeutic effect is mainly associated with the types of food intake and dietary patterns.

It has been demonstrated that different types and patterns of food intake can invoke different metabolic responses. In a controlled 24-h experiment, the effect of excess phosphorus intake from meat, cheese, whole grains, and phosphate supplementation was compared with that of normal phosphorus intake. The results showed that cheese could decrease serum PTH and bone resorption [132]. Dietary fermentable fibers [133], polyphenols [134], anthocyanins [135] and selenium-containing substances [136] can improve intestinal leakages by regulating oxidative pathways. In a 6-month clinical trial, daily supplement of blackcurrants that is rich in anthocyanins can mitigate bone for postmenopausal women through modulating the gut microbiota composition (increased *Ruminococcus 2*) and suppressing osteoclastogenic cytokines (decreased IL-1β, IL-2 and RANKL) [137].

Dietary patterns such as high-fat diet [138], high-glucose diet [139], and high-protein diet [140], have been demonstrated to impair the intestinal function, and need to be avoided to prevent the decline in GM abundance and the production of SCFAs. Some specific patterns, such as vegan diet, ketogenic diet, energy-restricted diet and anti-inflammatory diet [141], have a bidirectional effect on osteoporosis, and have been demonstrated to affect intestinal pH, GM structure and the production of SCFAs. The effects of the duration, the frequency, and the additional supplementation of calcium and vitamin in these dietary patterns still need to be investigated in clinical trials. Dietary patterns such as mediterranean diet, western diet, and eastern diet also have regional differences [141]. Essentially, these regional dietary patterns have different proportions of nutrients, which determine their roles in osteoporosis. In general, diets that are high fiber, high grains, low sugar and low fat have been recommended for preventing osteoporosis. Choosing a suitable dietary pattern based on health conditions of bone and intestinal, as well as keeping the dietary habits are important for preventing and treating osteoporosis. In addition, educational intervention programs and recommendations that provide patients with an in-depth understanding of osteoporosis may promote the therapeutic effect of these therapies [142,143].

### 4.2 Probiotics and prebiotics

Probiotics are defined as “live microorganisms that can provide health benefits to the host under a proper dosage” [144]. Prebiotics are defined as “food ingredients” that are indigestible and can be selectively used by microbes to improve the composition and activity of GM to provide health benefits to the host. For example, fructo-oligosaccharides (FOS) and galacto-oligosaccharides (GOS) are widely-used prebiotics [145]. The use of probiotics and prebiotics can affect GM by increasing GM diversity and SCFA production, selective growth of beneficial bacteria, modulation of immune responses, and so on.

Probiotics and prebiotics can effectively target intestinal barrier permeability. Evidence from various studies has shown that the consumption of probiotics and prebiotics is significant for the formation and maintenance of intestinal barrier as well as nutrient absorption. For example, *Lactobacillus reuteri*, a well-studied probiotic bacterium, has been shown to possess several beneficial properties. It has demonstrated the ability to delay the disruption of the intestinal epithelial barrier, decrease the *Firmicutes*/*Bacteroidetes* ratio and reduce bone loss of the femur and vertebral trabeculae [56]. In one study, Ewaschuk et al. supplemented wild-type (the control group) and IL-10-deficient mice (the experimental group with intestinal inflammation) with *Bifidobacterium infantis*. The results suggested that probiotics could normalize intestinal permeability by improving transepithelial resistance, increasing expression of ZO-1 and occludin, and decreasing expression of claudin-2 [146]. Besides, previous studies have shown that the addition of *Bacillus subtilis*, *L. rhamnosus*, heat-inactivated *Lactobacillus plantarum* and other probiotics can increase the VH/CD ratio, which can promote microbial colonization and improve nutrient absorption [147,148].

Numerous studies have demonstrated that probiotics, prebiotics, and their combined use (i.e., synbiotics) can prevent and treat osteoporosis by regulating different GM metabolites [149]. In one study, Tyagi et al. found that levels of butyrate and various bone formation marker increased in 10-week-old female mice after oral administration of probiotic *L. rhamnosus* Gorbach-Goldin (GG) for 4 weeks [112]. In another study, Rodriguez et al. evaluated the effect of 4-week supplementation of yacon flour (prebiotic), *Bifidobacterium longum* (probiotic), and yacon flour and *Bifidobacterium longum* (synbiotic) on the modulation of bone health, respectively. The results found that total anaerobic bacteria in the cecum, cecal concentration of propionate, and bone strength were higher for the rats in the supplementary groups than the control group. The combination of yacon flour and *Bifidobacterium longum* could also help increase the mineral content (Ca, Mg, and P) than other groups [150]. It has been reported by Lawenius et al. that the probiotic mix *Lacticaseibacillus paracasei* (DSM 13434), *Lactiplantibacillus plantarum* (DSM 15312) and *Lactiplantibacillus plantarum* (DSM 15313) can increase the bone mass in OVX mice [151]. Further research demonstrated that the role of the probiotic mix can increase concentration of propionate and acetate as well as crucial intermediates in SCFAs synthesis, namely succinate and lactate [152]. Lawenius et al. also developed a synbiotic composed of *Lactiplantibacillus plantarum, Levilactobacilus brevis, Leuconostoc mesenteroides, Pseudomonas fluorescens, Pichia kudriavzevii* and prebiotic dietary fibers based on their genome library (>4000 microbial strains). This synbiotic, codenamed SBD 111-A, is a defined microbial assemblage (DMA)™ food product, and it has exhibited improved therapeutic effect against osteoporosis in OVX mice by generating more SCFAs and vitamin K2 [153]. In one study, co-administering *Bifidobacterium lactis* Probio-M8 with conventional treatment (calcium, calcitriol) was more efficacious than conventional drugs treatment (calcium, calcitriol) alone in postmenopausal osteoporosis. It improved bone metabolism, as reflected by increased vitamin D3 levels as well as decreased PTH and procalcitonin levels in serum. Probio-M8 also affected the GM interactive correlation network, particularly SCFAs-producing bacteria (*Eubacterium*, *Blautia*, *and Ruminococcus*), suggesting a beneficial mechanism of probiotic adjunctive treatment [154].

Probiotics and prebiotics can also participate in immune regulation to treat osteoporosis. Sapra et al. found that supplementation with *L. rhamnosus* could attenuate bone loss by increasing the number of Treg cells and decreasing the number of Th17 cells [155]. Besides, *Lactobacillus acidophilus* could inhibit the secretion of osteoclastogenic factors (e.g., IL-6, IL-17, RANKL, TNF-α) and stimulate the secretion of anti-osteoclastogenic factors (e.g., IL-10, IFN-γ) during the maintenance of Treg-Th17 balance to promote bone formation [156]. These results reflected that the Treg-Th17 balance and related cytokines could be mediated by probiotics and prebiotics to regulate bone health. Besides, it has been suggested that reuterin and histamine produced by *L. reuteri* (ATCC PTA 6475) can suppress the activity of TNF-α and the expression of TRAP5 and RANKL to reduce post-menopausal bone loss [157].

Various clinical trials have also shown the therapeutic effect of probiotics for osteoporosis (See Table 2), such as *L. reuteri* [158,159], a mix of three *Lactobacillus* strains [160]. However, there are still several challenges. First, it is difficult for probiotics to colonize in the constantly renewing intestinal mucus [161]. Second, the effect of probiotics might be weakened by the acidic environment [162]. Therefore, synbiotics, which combine probiotics and prebiotics to take advantage of their synergistic effects and enhance survival rates, could be more effective in promoting microbial colonization and enhancing therapeutic effects than the role of a single probiotic [163]. Recent studies have also highlighted the utilization of engineered probiotic EVs as delivery system for osteoporosis treatment. For example, *Akkermansia muciniphila-*derived EVs have been designed to directly target bone cells, promoting osteogenesis and inhibiting osteoclastogenesis to preserve bone mass and strength in OVX mice [164]. However, natural EVs have several limitations, including suboptimal targeting capabilities and reduced therapeutic efficacy. To improve bone targeting, bone-targeting peptides were anchored on the *L. rhamnosus* GG-EVs membranes in a recent study, which can deliver intrinsic miRNAs to bone, enhancing osteoblast mineralization in OVX mice [165]. The development of probiotic EVs is still in its early stages. However, there is significant potential for personalized medicine, with EVs engineered to meet specific patient needs. Future research will likely concentrate on optimizing the delivery of these EVs and exploring their long-term effects on bone health.

**Table 2:** Targets and mechanisms of the gut microbiota for the treatment of osteoporosis (human experiments).

**Target/Intervention**	**Model**	**Duration**	**Results and related mechanisms**	**Ref.**
**GM structure**				
**Probiotics and prebiotics**	Elderly women with a good response (n = 10) and with a poor response (n = 10)	1 year	After one year of *Lactobacillus reuteri* (ATCC PTA 6475) supplementation, there was a notable reduction in inflammation and a significant increase in the GM richness for the good responders. Conversely, the poor responders exhibited distinct changes in microbial composition and function, characterized by the enrichment of *Escherichia coli* and the formation of its biofilm.	[158]
**Exercise**	Athletes (n = 40), healthy male controls (n = 46)	4 weeks	Compared with controls, athletes had a greater variety of micro-organisms within their gut, encompassing 22 different phyla.	[185]
**Exercise**	Previously sedentary overweight women (n = 19)	6 weeks	Enriched *Akkermansia* (a health-beneficial microbe) and depleted *Proteobacteria* (a health-detrimental microbe) were found in previously sedentary overweight women after 6 weeks of endurance exercise.	[184]
**GM metabolites**				
**Exercise**	Lean participants (n = 18 with 9 female) and obese participants (n = 14 with 11 female)	6 weeks	Endurance exercise can increase the levels of acetate, butyrate and other SCFAs in lean participants, but not obese ones.	[187]
**Immune regulation**				
**Exercise**	Young physically active (n = 15) and young physically inactive (n = 14), older physically active (n = 14) and older physically inactive (n = 17)	12 weeks	Exercise effectively reduced the activation of TLR4 and the production of pro-inflammatory cytokine IL-6 in both age groups.	[188]
**Hormone regulation**				
**Probiotic (Probio-M8) with calcium and calcitriol**	Postmenopausal osteoporosis patients with Probio-M8, calcium, calcitriol (n = 20) and with placebo, calcium, calcitriol (n = 20)	3 months	Co-administration of Probio-M8 improved bone metabolism markers, enhanced vitamin D3 levels, and reduced systemic inflammation and PTH levels, suggesting a beneficial effect on postmenopausal osteoporosis beyond conventional treatment.	[154]
**Exercise**	Postmenopausal women with aerobic exercise (n = 47) and with resistance exercise (n = 47)	12 weeks	Aerobic exercise could effectively reduce fat mass and body mass index (BMI), while anaerobic exercise could effectively increase muscle mass, estradiol levels, and BMD in comparison to aerobic exercise.	[192]

### 4.3 Fecal microbiota transplantation (FMT)

In FMT therapy, the feces of healthy humans or animals are treated to get the beneficial microflora for transplantation into recipient's gastrointestinal tract [11]. The therapeutic effect of FMT therapy on osteoporosis primarily involves the regulation of the intestinal barrier, GM structure, and immune response.

FMT can affect the barrier function by regulating occludin, claudin, and ZO-1. In a reverse experiment, the feces of 18-month-old SD female rats (the GM donors of osteoporosis) were transplanted to 3-month-old female rats (n = 16) by oral gavage three times a week during the 12 and 24 weeks of transplantation. The intestinal morphology including the sub-epithelial space and the villi structure, was impaired in 3-month-old female rats in week 24 [166]. FMT can also affect the GM structure. In the above reverse experiment, α diversity indexes of GM in FMT group were higher than those in control group in week 24. Regarding the β diversity, there was a substantial overlap between the FMT group and the GM donors in week 24. The result suggested that the enterotype of the donors could be transferred to the FMT groups in week 24 [166]. FMT therapy has been employed to treat osteoporosis. In an 8-week experiment, FMT therapy was performed on mice with osteoporosis induced by OVX by transplanting feces from healthy mice through daily gavage. The results demonstrated that FMT therapy can prevent bone loss by promoting the expressions of tight junction proteins (ZO-1 and occludin), increasing the SCFAs level (acetic acid and propionic acid), changing the GM structure (β diversity and abundance), and inhibiting pro-osteoclastogenic cytokines like TNF-α and IL-1β [167].

FMT therapy for osteoporosis is relatively few and mainly developed around animal experiments. More clinical trials are needed to identify mechanisms and improve therapeutic efficacy. In other diseases such as clostridium difficile infection [168], and inflammatory bowel disease [169], FMT therapy has been widely employed and clinical data have shown the potential on other targets like GM metabolites (e.g., SCFAs [170]) and hormones (e.g., IGF-1 [171]). However, the application of FMT therapy in the treatment of osteoporosis still needs to be further explored. For example, it is crucial to carefully consider the acceptance of donor sources and transplant methods for patients. The success and safety of FMT treatment depends on various factors related to donor selection and the transplantation process. In addition, some beneficial components of transplanted feces could gradually disappear after a few months, and additional transplantations or interventions are required to maintain the desired microbial balance.

### 4.4 Exercise

Physical therapy, or exercise, has been used for centuries to prevent and treat osteoporosis [172]. It has been suggested that different kinds of exercise can yield functional and beneficial outcomes in postmenopausal women with osteoporosis [173,174]. Mechanistically, regular exercise can increase the number of beneficial bacteria and reduce the number of harmful bacteria, promote absorption and metabolic function, and alleviate inflammation levels in osteoporosis [175]. Regular exercise can also induce mechanical stress on the bones, which is essential for maintaining bone health and stimulating the activity of bone cells [176]. The therapeutic effect of exercise on osteoporosis mainly depends on the types, duration, intensities, and frequency of the exercise [177,178]. However, it is worth noting that clinical trials often lack a standardized classification for the types of exercise. According to a review, exercise has been classified into aerobic exercise, progressive resistance training, weight-bearing impact exercise, multi-modal exercise training, and other modes [179]. Various effects of exercise on GM-derived mediators are shown in Table 2, which can enhance our understanding of the regulatory mechanisms involved in osteoporosis.

The effect of exercise on intestinal barrier depends on the types, duration, and intensities of the exercise [180]. For example, prolonged endurance exercise and high-intensity exercise can lead to impaired intestinal barrier function, and the mechanism could be related to systemic blood redistribution [181]. In this process, blood is mainly concentrated in the heart and skeletal muscles, which may lead to ischemia, hypoxia and other symptoms in the intestines. As a result, oxidative stress occurs and damages tight junction proteins and intestinal epithelial cells [182]. By contrast, it is reported that long-term aerobic exercise can positively maintain the intestinal villus morphology and repair intestinal permeability, which can be associated with the decreased expression of antioxidant enzymes, anti-inflammatory factors, anti-apoptotic proteins, and serum inflammatory factor levels [183]. For osteoporosis, exercise intensity and duration determine the state of intestinal barrier, local and systemic immune responses, which in turn influence the absorption of nutrients and the release of osteoclastogenic or osteogenic factors. Therefore, it is necessary for individuals to choose the appropriate amount and intensity of exercise according to their health conditions to prevent and treat osteoporosis.

Exercise can also adjust the types, diversity, and abundance of GM. For instance, Munukka et al. demonstrated that enriched *Akkermansia* (a health-beneficial microbe) and depleted *Proteobacteria* (a health-detrimental microbe) were found in previously sedentary overweight women after 6 weeks of endurance exercise [184]. McCabe et al. studied the effect of voluntary exercise on high-fat diet-induced bone loss in male mice aged between 6 and 20 weeks and suggested that voluntary wheel running could prevent high-fat diet-induced osteoporosis through the adjustment of the *Firmicutes/Bacteroidetes* ratio [34]. In a clinical trial, athletes exhibited a greater variety of gut micro-organisms that encompassed 22 different phyla than non-athletes. The study emphasized the combined impact of exercise and extreme diets on GM diversity [185]. The reduced harmful bacteria, increased beneficial bacteria, changes in GM structure and increased GM abundance can effectively improve SCFA production, absorption function and inflammation regulation, promoting bone formation.

Exercise can also stimulate the production of GM metabolites, which can lead to alterations in the SCFA profile. In animal experiments, running exercise has been observed to upregulate fecal butyric acid levels, which can be attributed to changes in the butyrate-producing bacterial population [186]. In a 6-week clinical trial conducted by Allen et al. the impact of exercise on GM was investigated in both lean and obese individuals with dietary control throughout the study. The results indicated that endurance exercise led to increased levels of acetate, butyrate, and other SCFAs in the lean participants. However, these changes were not observed in obese individuals. These findings implied that the exercise-induced alterations in the SCFA-producing capacity were largely dependent on obesity status, and the microbiome of lean individuals could be more responsive to exercise compared to overweight or obese ones [187]. The increased SCFAs caused by exercise could promote bone formation by the inhibition of osteoclast differentiation, promotion of osteoblast differentiation, and enhanced calcium absorption in bone metabolism.

Exercise can also mediate the immune system. A 12-week clinical trial was conducted to examine the impact of endurance and resistance exercise on previously sedentary older adults and younger adults. The results revealed that exercise effectively reduced the activation of TLR 4 and the production of pro-inflammatory cytokine IL-6 in both age groups [188]. Exercise can also mediate the levels of pro-inflammatory and anti-inflammatory cytokines. For example, aerobic, resistance, and combined trainings lasting from 6 weeks to 12 months were associated with lower levels of TNF-α, IL-6 and C-reactive protein in a meta-analysis study with 1510 postmenopausal women [189]. Moreover, exercise-induced elevation of intestinal IgA levels could enhance the resilience of mice against enteric pathogen infections. Additionally, it could also bolster their ability to resist colonization by symbiotic microbiota, thereby influencing the overall composition of GM [190,191]. For osteoporosis, decreased activation of TLR-related pathway (e.g. TLR 4, TLR 5), related inflammatory cytokines, and IgA levels are the potential mechanisms for osteoporosis. Further experiments are still needed to elucidate the relationship among exercise, osteoporosis, and the immune system.

Exercise can regulate the secretion of hormones, including estrogen, PTH, and glucocorticoids. For example, postmenopausal osteoporotic women (n = 94) took part in a 12-week exercise program, which studied the impact of aerobic and anaerobic exercises on estrogen level. The results indicated that aerobic exercise could effectively reduce fat mass and body mass index (BMI), while anaerobic exercise could effectively increase muscle mass, estradiol levels, and BMD in comparison to aerobic exercise [192]. Some previous results also demonstrated that 12-week resistance training significantly increased hormones (e.g., growth hormone, estrogen, parathyroid hormone, testosterone) [193,194]. Therefore, it can be inferred that moderate anaerobic exercise can promote the secretion of hormones, activate relevant GM-related receptors and release osteogenesis cytokines to promote bone formation.

Exercise intervention represents an effective strategy that can target multiple aspects of GM to treat osteoporosis. However, some issues remain to be addressed. Firstly, there is a lack of direct studies that examine and elucidate the mechanisms on how exercises affect GM that in turn will eventually lead to changes in bone metabolism, let alone how the variations in exercises can change the GM. Secondly, exercises being a systemic therapy, various factors such as a patient's age, gender, habits, and medical history can easily alter the responsiveness of GM to exercise. Currently various guidelines have been proposed to give patients exercise instructions, such as key loading characteristic, training principles, and evidence-based recommendations, to mitigate these possible interferences [179,195]. Furthermore, exercise has been combined with diet intervention [196], FMT [197], probiotics and prebiotics [198] to enhance their therapeutic effect on other diseases. Therefore, strategies based on exercise in combination with microbiome-based therapies are required for osteoporosis. Related mechanisms have to be investigated in animal experiments and clinical trials to validate the effectiveness of combination strategies. In addition, there are other emerging novel methods (e.g., warm exposure [199], GM-derived extracellular vesicles [200]) that are still at their infancy stage and await further development.

## 5 Conclusion and prospects

Recent research findings have shed new light on the causes of osteoporosis and shown GM as a promising therapeutic target for osteoporosis. Our review meticulously summarizes the potential links between GM and osteoporosis, as well as the prospective therapeutic avenues targeting gut microbiota for osteoporosis management, presenting a thorough and comprehensive review. Additionally, our work highlights a novel perspective on the combination of microbiome-based therapies and other non-invasive intervention for the treatment of osteoporosis.

In this review, we systematically concluded that GM impacted osteoporosis through two ways: intestinal barrier permeability and GM-derived metabolites (changes in GM metabolites, immune system, hormones). We then classify these changes as possible therapeutic targets of bone metabolism and highlight some associated promising microbiome-based therapies. Besides, the relevant mechanisms of some bi-directional regulation targets still require in-depth exploration. Additionally, several regulators in the gut–bone axis (e.g., immune cells and inflammatory factors) have been reported for other bone-related diseases (e.g., osteoarthritis), and their potential effects on osteoporosis require further elucidation.

We have further reviewed the current progress of three promising microbiome-based therapies, dietary intervention, FMT therapy, probiotics and prebiotics. Other conservative therapies such as exercise and warm exposure have also been discussed to show their effectiveness in treating osteoporosis. For microbiome-based therapies, large-scale clinical trials are needed in the future research to verify the effectiveness and safety of these microbiome-based therapies. While exercise as a conservative therapy, can be combined with dietary intervention, FMT therapy, probiotics and prebiotics, respectively. The combination can be employed as an integrated strategy to enhance the therapeutic effect of osteoporosis in the future study. In conclusion, by unraveling the mechanisms in the gut–bone axis and identifying new therapeutic targets, microbiome-based therapies have the potential to revolutionize the management of osteoporosis. These advancements may lead to personalized approaches that consider an individual's GM composition and offer more effective and targeted interventions, ultimately improving the quality of life for patients affected by osteoporosis.

## Declaration of competing interest

The authors have no conflicts of interest to declare.
